# A Review of the Therapeutic Efficacy and Safety of Human-Induced Pluripotent Stem Cell-Derived Cardiomyocytes in Preclinical Models of Subacute and Chronic Myocardial Infarction

**DOI:** 10.3390/jcdd13010042

**Published:** 2026-01-12

**Authors:** Kristen Callender, Godfrey Smith

**Affiliations:** School of Cardiovascular and Metabolic Health, University of Glasgow, Glasgow G12 8QQ, UK; 3050146c@student.gla.ac.uk

**Keywords:** human-induced pluripotent stem cell-derived cardiomyocytes, myocardial infarction, cardiac regeneration, animal model, ischemic heart failure

## Abstract

For the past decade, cell-based therapies have been the focus of research to investigate their potential to treat ischemic heart disease. The translation to human clinical studies depends on the demonstration of therapeutic efficacy and safety, particularly when transplanted in the subacute and chronic post-MI phase. A number of studies were identified that reported the effect of hiPSC-CMs on cardiac outcomes when transplanted at least 7 days post-myocardial infarction. The mean sample size of the published studies was 30 (±17) animals with a mean follow-up duration of 51 (±37) days. hiPSC-CM transplantation enhanced systolic function through augmented myocardial contractility, decreased infarct size, attenuated ventricular remodeling, and enhanced angiogenesis in the infarct and border zones in both small and large animal models. This effect was enhanced by co-transplantation with cells of vascular or adipose origin and is associated with high expression of VEGF in most studies. Despite this effect, transplanted hiPSC-CMs were structurally immature with limited survival at the endpoint. Epicardial delivery was associated with better efficacy outcomes and lower rates of arrhythmia. No study reported teratoma formation or immune rejection. From the current literature, there appears to be no consensus on the extent to which hiPSC-CMs improved systolic function, nor the degree to which this arises directly from integration of the new myocardium or from a paracrine-mediated mechanism. The nature of this paracrine mechanism and ways to improve the maturity and survival of implanted cardiomyocytes are issues that have yet to be resolved. In summary, while therapeutic benefit from cell therapy is clear, further research is required to establish whether the key mechanisms require a cellular component.

## 1. Introduction

Coronary artery disease (CAD) is the leading cause of heart failure (HF), accounting for 16% of all cardiovascular-related deaths globally [1]. CAD is characterized by a gradual accumulation of atherosclerotic plaque in the coronary arteries. When the plaque ruptures, it triggers thrombus formation within and downstream of the ruptured plaque and subsequent occlusion of the artery. Reduced or complete cessation of blood flow to the myocardium results in necrosis, otherwise known as a myocardial infarction (MI).

It was historically thought that the mammalian heart is a terminally differentiated organ with no capacity for endogenous repair. However, research shows that resident cardiac stem cells contribute to its innate, though limited, regenerative potential with a <1% annual rate of cardiomyocyte turnover [2]. Due to its restricted self-renewal capabilities, the heart cannot replenish the irreversible loss of cardiomyocytes from an MI and resorts to its default mechanism of repair–scar formation.

**Figure 1:**
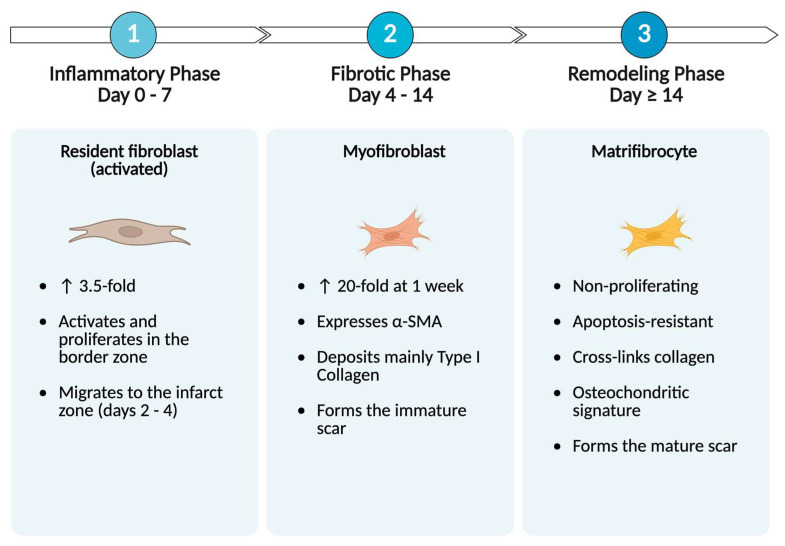
Wound healing and the process of scar formation post-MI in the adult human heart. This is divided into three phases: (1) inflammatory, (2) fibrotic, and (3) remodeling. Each stage is denoted by a specialized phenotype of the resident injury-responsive fibroblasts. Neoangiogenesis begins at ~4 days and its duration depends on the extent of injury. Note, this is subject to interindividual and interspecies variation. In murine models, scar maturation has a shorter duration, and angiogenesis begins at ~day 2 [4]. Scar formation begins around day 7 and day 14 post-MI in small and large animal models, respectively [4]. Myofibroblasts deposit both Type I and III collagen but Type I predominates in the infarct zone, otherwise called replacement fibrosis. Symbol ↑ represents increase. Created in BioRender. Callender, K. (2026) https://BioRender.com/xtj62nd, accessed on 7 January 2026.

Scar formation begins during the inflammatory phase of post-MI healing, when resident fibroblasts in the ischemic border zone (BZ) are activated and migrate to the infarct zone (IZ). The fibrotic phase is characterized by their conversion to a transient *α*-SMA-positive myofibroblast phenotype. Myofibroblasts deposit predominantly Type I collagen in the IZ, forming an immature scar. In the remodeling phase, myofibroblasts transform into a quiescent phenotype called matrifibrocytes, which stabilize the scar through their osteochondritic signature and collagen cross-linking [3] (Figure 1).

**Figure 2:**
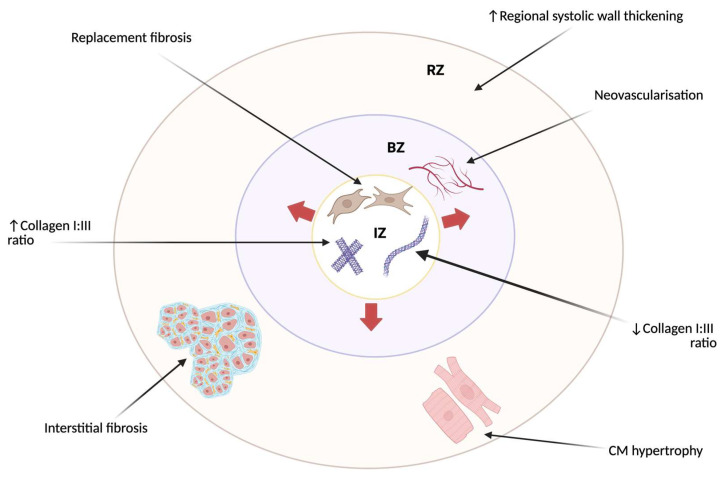
Features of remodeling in the three zones of the myocardium post-MI. The infarct zone (IZ) is the focus of necrosis. The border zone (BZ), also known as the ischemic penumbra, is closest to the IZ. Here, cells may still be viable and salvageable if re-perfused. The remote zone (RZ) is farthest away from the IZ, where structural and functional changes compensate for loss of function in the IZ. Red arrows represent infarct expansion. After an MI, the damaged myocardium in the IZ is replaced with fibrotic tissue which forms the scar. This is known as replacement fibrosis and is distinct from interstitial fibrosis, which refers to the accumulation of excess collagen and ECM between the cardiomyocytes located in the RZ. Increased deposition of collagen Type I and III occurs, but with an alteration of the ratio of ‘good’ collagen (Type I) and ‘bad’ collagen (Type III). Collagen Type I predominates in replacement fibrosis, but Type III predominates in BZ fibrosis. The collagen I–III ratio in healthy myocardium is usually 5:1 [7]. Regional wall thickening in the RZ increases after an MI to compensate for the decreased thickening in the ischemic area [8]. This is reflected as an increase in strain parameters or radial dyssynchrony, as shown in a few studies. Symbols ↑ and ↓ represent increase and decrease. Created in BioRender. Callender, K. (2026) https://BioRender.com/0nq91yq, accessed on 7 January 2026.

Infarct scar tissue is vascularized, metabolically active, and restores the structural integrity of the ventricle [5]. However, myofibroblasts and matrifibrocytes lack the inherent contractile properties of normal cardiomyocytes and are resistant to apoptosis [6]. Physiological hypertrophy of healthy cardiomyocytes compensates for the loss of cardiomyocytes in the IZ (Figure 2). Subsequent thinning of the ventricle occurs under sustained mechanical stress and profibrotic factors [6]. This persistent maladaptive remodeling leads to HF and electrical dysfunction.

Current evidence-based medical therapy reduces mortality in patients with ischemic HF [9]. While these therapies attenuate progression and symptoms, they cannot reverse ischemic myocardial injury, much less replace scar tissue. This shortcoming fueled research into the adjunctive use of cell-based therapies in post-MI repair.

## 2. Prior Research on Cell-Based Therapies

The early use of skeletal myoblasts (SMs) in myocardial repair was based on the central idea of cardio-myogenesis as its sole mechanism. Menasché P et al. described the first autologous transplantation of SMs in the infarcted myocardium of an elderly male, resulting in a modest improvement in the left ventricular ejection fraction (LVEF) and functional status [10]. Preluded by mixed preclinical outcomes where failed integration and differentiation provoked arrhythmogenicity [11,12], studies using SMs were aborted. The focus shifted to cell-based strategies aimed at direct and indirect regeneration, forming the bedrock for the ‘first generation’ of cell types. These include bone marrow mononuclear cells (BMMNCs) and their subpopulations: mesenchymal stem cells (MSCs), endothelial progenitor cells (EPCs), and hematopoietic stem cells (CD134^+^/CD133^+^).

**Figure 3:**
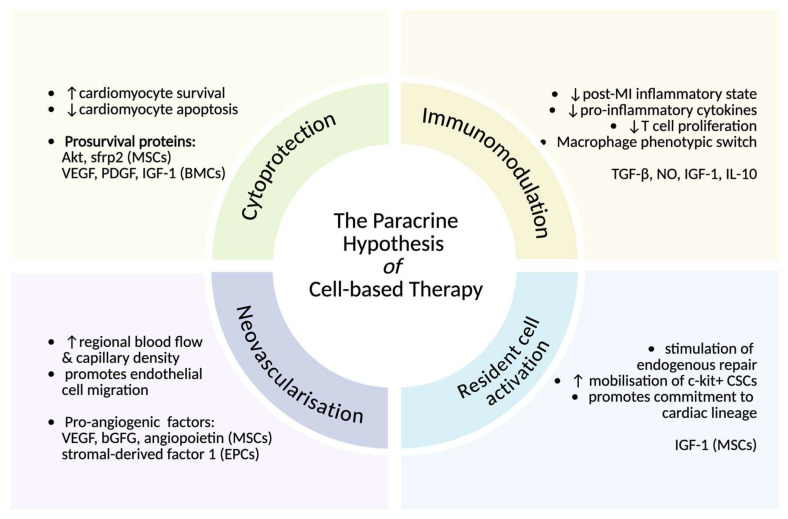
Paracrine mechanisms of post-MI stem cell repair. Evidence suggests that stem cells exert their reparative effects through an indirect mechanism that involves the secretion of paracrine factors acting on resident cells as opposed to the direct remuscularization of the heart [16]. Through this paracrine mechanism, stem cells influence cardiomyocyte survival, modulation of the immune response, neovascularization, and stimulation of endogenous repair. (Symbols ↑ and ↓ represent increase and decrease). Created in BioRender. Callender, K. (2026) https://BioRender.com/10skfnl, accessed on 7 January 2026.

MSCs are the most promising class, with evidence of successful integration, differentiation, and infarct size reduction in animal models of chronic ischemic cardiomyopathy [13,14,15]. These effects are mediated by indirect paracrine mechanisms, as illustrated in Figure 3. BMMNCs significantly improved LVEF and decreased the risk of major adverse cardiovascular events (MACEs) long term; this effect was enhanced by culture periods of >1 week and cell counts of ~10^8^ [16]. MSC use is challenged by poor engraftment rates (6–12%) [16] and marked heterogeneity in tissue sources and delivery routes [17], accompanied by a focus on their effect in the acute post-MI period [18].

‘Second-generation’ cells are genetically and chemically ‘engineered’ in vitro to enhance paracrine activity, engraftment, plasticity, and survival, making them superior to ‘first-generation’ cells [19]. These include cardiac stem cells (cardiosphere-derived cells and cardiac progenitor cells), pluripotent cells (embryonic stem cells and induced pluripotent stem cells), and cardiopoietic cells.

Cardiac stem cells (CSCs) are sourced from cardiac biopsy, giving them the clear advantage of being a pre-existing ‘cardiogenic phenotype.’ In preclinical studies, CSC therapy caused a 10.7% increase in LVEF, with greater effect observed in small animal models [20]. The intra-coronary administration of cardiosphere-derived cells (CDCs) significantly reduced scar mass and increased viable heart mass and regional contractility in patients up to 3 months post-MI [21]. However, CSCs have limited yield and engraftment rates and transition to clinical trials outpaced the effective address of these challenges.

Embryonic stem cells (ESCs) and induced pluripotent cells (iPSCs) are pluripotent and are capable of differentiating into any cell type. This comes with a tumorigenic risk when administered in an ‘undifferentiated state’. To overcome this hurdle, in vitro pre-differentiation committed to a cardiac lineage was developed. In a landmark preclinical study, ESC-derived cardiomyocytes integrated and functionally matured, resulting in the improvement of echocardiographic parameters [22]. Similarly pre-differentiated ESC-derived ISL1^+^ cardiac progenitor cells formed a ventricular patch months after transplantation in a non-cardiac environment (the subscapular portion of a rat kidney) [23]. In patients with severe ischemic HF, epicardial delivery of ESC-derived CPCs was feasible, safe, and yielded modest wall motion enhancements in the treated segments after 1 year [24].

The discovery of iPSCs [25] provided an appealing solution to the immune rejection and ethical concerns associated with ESCs [26]. iPSCs are reprogrammed from a patient’s own somatic cells by expression of key pluripotency factors (OCT-4, c-myc, KLF4, SOX-2, and Nanog) using virus-based or non-integrating vectors [27]. They can be pre-differentiated into cardiomyocytes in vitro.

iPSC-CMs adopt a fetal-like state, making them morphologically and electro-physiologically different from adult cardiomyocytes. They are rounder, smaller, and mononuclear with disordered sarcomeres and no T-tubules, whereas adult cardiomyocytes are elongated with two or more nuclei and well-organized sarcomeres and T-tubules [28]. iPSC-CMs express ssTnI (a myofibril troponin isoform expressed in fetal myocardium), whereas adult cardiomyocytes express cTnI. Moreover, iPSC-CMs depend on glucose for metabolism, whereas adult cardiomyocytes derive energy from fatty acids [28].

Though it was hoped that these stem cells could directly remuscularize the heart through trans-differentiation to functional cardiomyocytes, there is minimal evidence of post-transplantation survival [29]. One of the main reasons proposed for this poor engraftment is the exposure of the transplanted cells to the harsh ischemic and oxidative environment in the post-MI inflammatory phase [30]. Undifferentiated iPSCs enhanced myocardial contractility and halted ventricular remodeling in acute MI [31], but evidence regarding the effect that iPSC-CMs have on those outcomes when transplanted outside the inflammatory phase is unknown. This comprehensive review will synthesize the current evidence regarding the therapeutic efficacy and safety of human iPSC-CMs when transplanted in the fibrotic or remodeling phase of post-MI healing in animal models.

## 3. Studies Investigating the Therapeutic Use of Derived Cardiomyocytes in the Context of Ischemic Heart Disease

### 3.1. General Characteristics

A summary of the featured studies is presented in Table 1 [32,33,34,35,36,37,38,39,40,41,42,43,44,45,46,47,48,49,50,51,52,53,54]. There was a balanced representation of small and large animal models. The mean sample size was 30 ± 17 (SD), ranging from 10 to 72 animals. Mean post-transplantation follow-up periods were 51 ± 37 (SD) days with the shortest being seven days [42] and the longest, six months [51]. Transplantation occurred on average Day 20 ± 16 (SD) post-MI. The longest time from MI induction to the end of follow-up was 210 days—a 28-day period from MI to transplantation and another 182 days of post-transplantation follow-up [51].

The most common method of MI induction was permanent ligation of the left anterior descending artery (LAD), either by suture [34,35,36,38,39,42,44,45,48], ameroid constrictor [37,40,41,43], or a combination thereof [46]. Other methods included permanent ligation of the left coronary artery (LCA) [34,44,47,53], ischemic reperfusion (I/R) injury [32,33], and cryoinjury to the LV anterior free wall [49,50,52]. Li et al. was the only study that used the sequential balloon sponge embolization technique, where portions of the LAD were occluded for five minutes in 60 s intervals [54].

Most studies employed an epicardial delivery route [34,35,37,38,39,40,41,43,44,46,47,50,51,52,53]; less commonly used routes were hydrogels [40], spheroids [33,49], and a cells-only approach [32,36,45,48,54]. In a few studies, hiPSC-CMs were co-transplanted with another cell type such as hiPSC-derived endothelial cells (hiPSC-ECs) [32,41,50,53], hiPSC-derived mural cells (hiPSC-MCs) [41,53], human neonatal dermal fibroblasts (hNDFs) [34,51], or human adipose mesenchymal stromal cells (hADSCs) [33]. Sun X et al. delivered hiPSC-CMs with rat adipose tissue micro-vessels in a Type II Diabetes Mellitus-infarcted rodent model [45]. All studies transplanted cells at quantities equal to or greater than 10^6^. Spheroids had the highest purities (>99%) [33,49].

### 3.2. Effect of Transplantation of hiPSC-CMs on Myocardial Function and Structure

Most studies observed a significant increase in LVEF [32,33,35,36,37,38,39,40,41,42,44,45,46,47,48,49] and fractional shortening (FS) from baseline [33,36,39,41,42,45,47,53] (Table 2). Similarly, a significant increase in the fractional area of change (FAC) was observed as early as 2 weeks and sustained until 2 months post-transplantation of a hiPSC-CM cell sheet combined with hiPSC-MCs and ECs [53]. Dual intramyocardial injection of hiPSC-CMs and hiPSC-ECs caused a significant increase in EF (8.9 +/− 1.3% *p*: 0.0005) by 28 days but was not significant when CMs were administered alone (1.9 +/− 4.1% *p* = 0.65) [32]. The LVEF was significantly lower in a patch-treated porcine model than the control group at 6 months (49.3 +/− 3.5% vs. 55.4 +/− 2.2% *p* < 0.05) [51]. In one instance, larger spheroids containing 6 × 10^7^ CMs showed a sustained improvement in FS at 12 weeks (7.4 +/− 1.3% *p* < 0.01), whereas smaller spheroids containing 2 × 10^7^ CMs showed transient improvement at 4 weeks but lost significance by 12 weeks (5.1 +/− 1.1% ns). Weinberger et al. reported no change in FAC with patches containing ECs alone compared to a 31% increase observed in composite patches of hiPSC-CMs and ECs [50]. A significant increase in the FAC was only observed with patches containing higher doses (12 × 10^6^ CMs) but not with lower doses (4.5–8.5 × 10^6^ CMs) [52].

Most studies observed no significant decrease in LV end-diastolic diameter (LVEDD) [32,33,35,36,38,40,42,45,46,50,54] but saw a significantly smaller LV end-systolic diameter (LVESD) at the endpoint [32,35,36,38,43,44,45,46,47]. There was a notable trend of improved systolic function without significant changes in LV end-diastolic volume (LVEDV) from baseline [32,33,36,45,47,49]. The positive rate of pressure change with time (+) dP/dT) was significantly increased in three studies [45,47,49], whereas one study observed no significant change [34]. Kawaguchi et al. demonstrated a significant increase in (+) dP/dT with spheroids but not with single hiPSC-CMs when compared to the control [49].

Three studies [34,45,47] measured and reported significant decreases in tau, whereas one study demonstrated a significant increase from baseline though it was not significantly different to the untreated controls [51]. Lancaster et al. was the only study to report a significant increase in diastolic parameters E/e’ and e’/a’ compared to the control [34].

One study explored the effect on end-systolic elastance (Ees) and the end-diastolic pressure–volume relationship and found a significant improvement in infarcted pigs treated with a composite patch at 6 months [51]. This same study demonstrated a reduction in myocardial edema at 6 months follow-up, reflected by a significant decrease in T2 relaxation time on CMR [51]. Patch-treated pigs had a significant change in total activity and an increase in heart rate response to exercise when put on a treadmill [51]. Four studies showed a significant increase in global and regional circumferential strains in transplanted animals compared to the control [41,46,48,54].

### 3.3. Effect of Transplantation of hiPSC-CMs on Infarct and Scar Size

Of twenty-three studies, seven reported infarct size [32,33,39,45,50,51,54] but only four measured the change in infarct size in comparison to a cell-free control [32,39,45,54]. Two co-transplantation studies showed no significant reduction in infarct size compared to the control at 28 days when hiPSC-CMs were intramyocardially injected alone but showed significantly reduced infarct size when co-transplanted with hiPSC-ECs or micro-vessels [32,45]. Similarly, Li et al. showed no significant difference in infarct size after a post-transplantation follow-up of 84 days [54]. Only one study using cell sheets reported significant reductions in infarct size at 28 days [39] (Table 3).

Three studies explicitly reported scar area reduction. Coincidentally, these were all large-animal models with transplantation at two to four weeks post-MI: two spheroid grafts [33,49] and one patch [46]. Of the two spheroid studies, one of them reported a significantly smaller proportion of scar area at eight weeks compared to the cell-free control (12.4 +/− 2.1% vs. 17.4 +/− 3.9% *p* < 0.001) [49]. The other did not report any comparison to the control but reported no significant difference between spheroid sizes [33]. Even though a hiPSC-CM seeded patch reduced scar size in an infarcted porcine model at 12 weeks, it was not significant [46].

### 3.4. Effect of Transplanted hiPSC-CMs on Outcomes of Survival, Retention, and Structural and Functional Integration

Studies showed advanced sarcomere structures with increased sarcomere length and predominance of the MLC2v phenotype but grafted CMs were often disorganized and smaller than host CMs with immature mitochondria and dispersed connexin-43 expression [32,33,38,43,45,48,49,50,52]. Two studies identified pancadherin in their grafts [32,48]. One study showed clear expression of connexin-43, thickened myofibrils, mitochondria with many lamellar cristae, adherens junctions, and desmosomes between cells at 12 weeks. This was a cell sheet coated with fibronectin, but little fibronectin was detected at follow-up. Instead, new extracellular matrix (ECM) components such as collagen Type IV, perlecan, and desmin were identified [35].

Twelve studies demonstrated evidence of transplanted hiPSC-CMs at the end of the follow-up periods: at 4–5 weeks [36,39,41,42,44,45,48,50,52], 8 weeks [40,49], and 3 months [43]. However, transplanted cell numbers were relatively low. Querdel et al. showed a significant increase in survival rate with increasing dose [52]. Cell sheets cultured under dynamic conditions survived in more rats (n = 7 of 10) than sheets cultured under static conditions (n = 3 of 10) [44]. At three months, 57 +/− 10% of SPIO-labeled hiPSC-CMs from cell sheets reinforced by omentum flaps were identified compared to 25 +/− 5% in the sheet-only group [43]. No cells survived at the end of the follow-up period in two studies [34,49].

Only two studies assessed the functional integration of transplanted hiPSC-CMs [38,50]. Samura et al.’s laminin-conjugated cell sheet showed spontaneous beating with rat CMs in a 4:1 ratio of calcium transient using a genetically coded calcium indicator (GCamP) [38]. Weinberger et al. demonstrated evidence of epicardial electric coupling in the graft region, uniform subendocardial signal amplitude across the infarct, and transmural electrical coupling in the IZ of patch-treated guinea pig hearts with entrainment to a pacing cycle length (PCL) of 120 to 250 ms with beat-to-beat variability (normal guinea pig PCL is 200–300 ms). However, this was also associated with shorter action potential duration and faster transmural conduction in the remote myocardium [50].

Cell washout occurred after the transplantation of a composite hiPSC-CM/EC patch where cells were found in the spleens and lungs of six infarcted guinea pigs [50]. In contrast, intramyocardially injected cells were confined to the heart after 4 weeks [36]. Significantly higher retention rates were observed in rats who received CMs dissolved in higher concentrations of hydrolyzed gelatin compared to CMs alone (20% HG: *p* < 0.0001, 10% HG: *p*= 0.0018) [42].

### 3.5. Effect of Transplanted hiPSC-CMs on In Vivo Remodeling of Native Myocardium

#### 3.5.1. Changes in Native CM Size and Fibrotic Area

Native CM hypertrophy in the BZ and remote zone (RZ) was significantly decreased in six studies [38,39,40,41,44,49]. There was no significant difference in CM size in the RZ between pigs that received spheroids and those that received single CMs (*p* = 0.769) [49]. Fibrotic area was quantified in the RZ in five studies [35,38,40,44,46] and was found to be significantly less in the treatment groups compared to controls. In contrast, Miyagawa et al. reported no significant difference [46]. Collagen-positive areas in the BZ were significantly decreased in animals that received hiPSC-CMs co-transplanted with rat adipose-derived microvessels than CMs alone and the acellular control [45]. Yokoyama et al. noted no significant difference in the fibrosis of the whole cardiac tissue between fibronectin-coated hiPSC-CMs and the control in a rat MI model [35]. Interstitial fibrotic reduction was enhanced by dynamic culturing (*p* = 0.0035 compared to static culturing) [44] and laminin conjugation (*p* = 0.03 compared to laminin-free) [38]. In the five studies that measured fibrotic area but did not specify the location, all but one [36] reported a significant reduction compared to the control [39,41,53,54].

#### 3.5.2. Changes in Vascular Density

Four studies observed significant neovessel formation in the transplanted area of grafted animals [32,39,43,45]. In a cryoinjured guinea pig infarction model, capillary density in the graft was significantly lower than the host myocardium despite receiving the highest dose of hiPSC-CMs (12 × 10^6^ CMs) [52]. Reinforcement of a cell sheet with a pedicled omentum flap resulted in a significantly enhanced graft capillary abundance compared to those without the flap [111 ± 35 units/mm^2^ vs. 51 ± 22 units/mm^2^ *p* < 0.05] [43]. Similarly, co-transplantation hiPSC-ECs or hADSCs enhanced graft vascular density compared to hiPSC-CMs alone [32,39].

Vascular density in the BZ was also significant regardless of construct or cell type [32,35,37,38,39,40,41,46,49,50]. In a rodent model, CM-only sheets did not significantly enhance BZ vascular abundance; however, when combined with hADSCs, arteriolar and neovessel abundance increased (*p* < 0.01) [39]. An hiPSC-CM sheet was superior to MSC- or SM-derived cell sheets in enhancing neoangiogenesis in both the IZ and BZ at two months post-transplantation in a porcine MI model [37]. Two studies observed significant vascular abundance in the BZ associated with adjuvant ECs [32,50]. There was no evidence of significant enhancement of vascular density in the RZ [32,49].

#### 3.5.3. Changes in Cytokine Expression

Transplantation of hiPSC-CMs caused significant increases in the expression of the following pro-angiogenic cytokines: VEGF [34,35,38,40,43,44,46,49], ANG-1 [34], b-FGF [38,40,43,46,49], HGF [35,44], SDF-1 [38,43], and PDGF [38,49]. Infarcted pigs treated with hiPSC spheroids highly expressed VEGF (~5–10 × 10^3^ pg) in addition to smaller quantities of other cytokines [49]. Two studies assessed in vitro cytokine expression before transplantation: Ishida et al. showed that hiPSC-CMs, SMs, and MSCs expressed cytokines IGF-1, VEGF, and SDF-1 similarly with no significant difference [37] and proangiogenic cytokine expression was significantly higher in cell sheets with adjuvant hADSCs compared to hiPSC-CMs alone (*p* < 0.001) [39]. All studies that reported cytokine expression demonstrated significant increases in vascular density in vivo.

### 3.6. Safety and Tolerance

#### 3.6.1. Incidence and Characterization of Post-Transplantation Arrhythmia After the Transplantation of hiPSC-CMs

Of the seven studies that assessed post-transplantation arrhythmogenicity, five were intramyocardial [spheroids (n = 2), cells (n = 3)]. Four of them reported the incidence of arrhythmia in large animal models [32,33,49,54]. Arrhythmias were characterized as ventricular tachycardia (VT) or paroxysmal supraventricular tachycardia (pSVT) and peaked around day 7 [33], 14 [33], and 21 post-transplantation [32,49]. Larger spheroids (6 × 10^7^ CMs) resulted in 13 episodes of sustained VT compared to 1 episode observed with smaller spheroids (2 × 10^7^ CMs) [33]. Three studies reported no post-transplantation arrhythmias at the end of the follow-up period [34,36,46]. One study reported a high incidence of sustained VT despite initiation of anti-arrhythmic therapy (24 mg/kg of amiodarone) five days before transplantation [32].

#### 3.6.2. Evidence of Tumor Formation After the Transplantation of hiPSC-CMs

Of the 23 studies, six studies assessed in vivo tumorigenicity [37,40,46,49,50,53] and observed no teratoma formation (Table 2 and Table 3). Four of them were immunocompetent porcine models. Data were not shown for one study [37]. Evidence of post-transplantation proliferation using ki67 immunostaining was demonstrated in five studies [39,45,48,50,52]. Only two of these studies reported the ki67 index, which averaged 7.71 +/− 2.51% [45,48]. Cell cycle activity averaged 10.05% before transplantation and increased by 16.1 +/− 4.6% (mean of two studies) in two guinea pig MI models [50,52]. One study [46] assessed tumorigenicity by measuring in vitro levels of LIN28 and found low levels in the patch 30 days after differentiation despite ~20% residual undifferentiated cells.

#### 3.6.3. Evidence of an Immune Response, Immune Rejection, and Immunosuppressive Regimens Used

Of the 16 studies that used immunocompetent animal models, 13 of them received immunosuppression [32,33,36,37,40,41,43,46,48,49,50,52,54]. Three studies using immunocompetent animals did not use immunosuppression [34,47,51]. One study reported the use of immunosuppression but did not specify the drug regimen [41]. Most of the studies used a triple-drug therapy which included a corticosteroid (methylprednisone or prednisone), tacrolimus or cyclosporine A, and mycofenolate mofetil [37,43,46,49,54]. Abatacept replaced mycofenolate mofetil in two NHP models [32,33]. Daily cyclosporine A (20 mg/kg) [48] or tacrolimus (0.6 mg/kg) [40] was used as monotherapy in two studies. Immunosuppression was typically initiated 1–3 days before or on the day of transplantation until sacrifice; however, Biagi et al. initiated it 48 h after MI, which was approximately 35 days before transplantation [48].

CD45+ leukocytes and CD3+ lymphocytes invaded the transplanted area of a triple immunosuppressed porcine MI model at two weeks post-transplantation but the response attenuated by four weeks [49]. Another study found significantly more frequent patchy infiltrates in treated rats compared to the control. However, 50% of these infiltrates were classified as Grade 0–1 [48]. A study that did not use immunosuppression demonstrated a significant increase in IgM levels (*p* = 0.0462) at 21 days post-transplantation and detectable graft-specific IgG levels from one week post-transplantation with a steady rise by week 7 (*p* = 0.0069) [47]. Despite this, none of the 23 studies reported evidence of immune rejection.

## 4. General Conclusions

### 4.1. hiPSC-CMs Cause a Dose-Dependent Long-Term Improvement in Myocardial Contractility Post-MI

Across the board, there was a significant improvement in systolic function with little to no change in LVEDV. This means that the pumping efficiency of the heart was increased without a change in filling volume and could suggest increased contractility as the driving force behind this improved cardiac output. Load-dependent parameters such as EF, FS, and FAC were routinely used to measure systolic function. These values are influenced by afterload and preload and are unreliable measures of inotropy. More sensitive inotropic parameters are Ees, (+) dP/dT, and preload recruitable stroke work (PRSW). (+) dP/dT was significantly higher after four weeks post-transplantation but not for shorter follow-ups. Interestingly, there was one case of increased inotropy (measured by Ees) but decreased stroke volume and EF at six months follow-up with a significant concurrent improvement in tau [51]. An increase in tau equates to increased stiffness of the LV, which could inadvertently decrease cardiac output over time. There was a notable dose-dependent effect on systolic recovery with spheroids. Larger spheroids resulted in sustained improved cardiac function, whereas smaller spheroids caused transient improvement [33].

### 4.2. Co-Transplantation with Auxiliary Cells Enhances the Post-Transplantation Therapeutic Effect of hiPSC-CMs

There was a consistent augmented therapeutic effect of hiPSC-CMs co-transplanted with auxiliary cells, particularly of vascular (mural cells and endothelial cells) [32,41,50,53] or adipose origin. Even though this could imply that hiPSC-CMs need support in vivo, evidence shows that auxiliary cells cannot attain the same effect independently [50]. The two studies that showed improvements in all key therapeutic efficacy outcomes were both co-transplantations: the intra myocardial injection of hiPS-CMs with micro vessels from rat adipose tissue [45] and a composite cell sheet of hiPSC-CMs and hADCS. Recent experiments showed that hADSCs exhibited a paracrine influence on the structural and electrophysiological properties of hiPSC-CMs in coculture [55]. Co-transplantation of hADSCs also enhanced vascular density in the graft itself, which likely contributed to its survival by the endpoint. This relationship between neo-angiogenesis and adipose-derived stem cells is not new. The literature shows that these cells promote angiogenesis via a hypoxia-driven differentiation into ECs [56] and secretion of pro-angiogenic soluble factors such as bFGF and adiponectin [57]. bFGF is one of the cytokines assessed frequently in these studies and cardiac adiponectin has been found to have cardioprotective effects after I/R injury through attenuation of oxidative stress [58]. More importantly, the only solid evidence of functional integration was reported in a study using a patch supplemented with ECs [50].

### 4.3. Pre-Transplantation Vascularity Is Crucial to the Long-Term Engraftment and Survival of hiPSC-CMs In Vivo

One of the main reasons for poor post-transplantation engraftment of hiPSC-CMs is lack of vascularization in the infarcted area. This review supports evidence that hiPSC-CMs stimulate neovascularization in vivo and shows that measures used to enhance graft vascularity prior to transplantation are just as crucial. An engraftment of 73%—which far exceeds the usual 13% proposed in the literature [45]—was reported in a study showing anastomosis of graft vessels with the native vasculature [45]. Furthermore, a cell sheet reinforced with an omental flap transplanted in a pig had the longest survival of 3 months [43]. Omentum plays a valuable role in complex wound healing due to its robust vascularity and pro-angiogenic and anti-inflammatory properties [59]. This highlights the crucial role of vascularization in the maturity and viability of hiPSC-CMs post-transplantation.

Vascularity remains a significant issue with cell sheets and patches due to limited diffusion of nutrients and oxygen to the transplant site. This is likely caused by their thickness, especially when applied in layers, as seen in a few studies [37,40,41,44,53]. Four studies employed methods to address this: (1) using a fibrin scaffold (a structural framework that guides the formation of new blood vessels) [39], (2) a dynamic culture in a bioreactor (which provides a continuous supply of oxygen and nutrients to the cells) [44], (3) reinforcement with an omentum flap [43], and (4) supplementing with ECs [50]. All these studies showed remarkable rates of survival.

### 4.4. hiPSC-CMs Exert In Vivo Paracrine Effects in the Short Term That Are Sustained in the Longer Term

Enhanced contractility without robust evidence of survival or structural and functional integration can only be explained by an indirect mechanism of remuscularization. Furthermore, changes occurred in healthy myocardium remote to the transplantation site. In a study by Ishida et al., MSCs and hiPSC-CMs produced similar levels of three cytokines (iGF-1, VEGF, and SDF-1) before transplantation, suggesting that both cell types may share similar paracrine properties [37] (Figure 3).

VEGF is a protein that plays a key role in angiogenesis and was the most common cytokine across the studies. Markedly high levels of expression were observed at the endpoint in a study that reported engraftment failure [49]. Transient transfection of hiPSC-CMs with modified VEGF RNA caused significant improvement in systolic recovery as early as two weeks post-transplantation, along with prolonged survival and enhanced expression of connexin-43 and the formation of endothelial tubules in vitro [60]. Injection of modified VEGF mRNA alone resulted in the enhancement of sensitive inotropic parameters (PRSW and (+) dP/dT) in a subacute post-MI porcine model [61]. Early research revealed a direct effect of VEGF on contractility through modulation of calcium transients via the VEGF-FLT1-PLCγ1 pathway which is independent of its impact on angiogenesis [62]. This could imply that the enhanced contractility seen with hiPSC-CMs may be directly mediated by VEGF.

### 4.5. hiPSC-CMs Attenuate Post-MI Ventricular Remodeling

Transplantation of hiPSC-CMs attenuated remodeling through multiple mechanisms: decreasing regional systolic thickening [37,53], decreasing akinetic length [42,53], decreasing native CM hypertrophy in the BZ and RZ [38,39,40,41,44,49], reducing myocardial wall stress in the IZ [37], decreasing interstitial fibrosis [35,38,40,44,46], and enhancing neovascularization and reducing collagen content in the BZ [45]. This is clinically relevant as attenuation of all these processes reduces infarct expansion, a crucial stage in the development of thinning and disproportionate dilation of the infarcted area [63]. Moreover, most of these changes occur in the BZ, the main target of early reperfusion therapy in clinical practice. Reduction in CM hypertrophy also attenuates the development of electrical dysfunction that predisposes to post-MI arrhythmias.

### 4.6. Therapeutic Efficacy and Safety of hiPSC-CMs Are Influenced by the Nature of the Construct

Both epicardial and intramyocardial delivery of hiPSC-CMs enhanced vascular density and systolic function. Post-transplantation arrhythmias were more likely to occur with intramyocardial injections, whether it be spheroids or single cells. Graft survival was higher with epicardial delivery, and this effect was primarily driven by cell sheets. Reduction in infarct or scar size was observed more in recipients of intramyocardial hiPSC-CMs. The lack of reporting of infarct/scar size with epicardial delivery may be due to the failure of distinguishing between the transplant border and the scar border, as was the case in the study by Weinberg et al. [50].

Cell sheets had the highest rate of all therapeutic efficacy outcomes and were associated with enhanced maturity and were more likely to attenuate remodeling of the native myocardium. The superiority of cell sheets may be due to a couple of reasons: epicardial delivery reduces the risk of further damage and inflammation to the application area and they can be supplemented with components that support structural integrity, such as an ECM.

Spheroids have a hypoxic core that makes them ischemia-resistant in vivo [64]. However, engraftment in a porcine model failed with this construct but succeeded in a rat model of the same study [49]. With no evidence of immune rejection, failure could have been caused by spheroid size. Evidence suggests that the larger the spheroid, the less availability of nutrients, resulting in increased apoptotic marker expression [65]. This study delivered 100 million CMs to the porcine model but 3 million CMs to the rat. Another spheroid study delivered CMs between 20 and 60 million in a large animal model with no reports of failure [33].

### 4.7. Transplantation of Pre-Differentiated hiPSC-CMs Is Safe with a Low Incidence of Immune Rejection or Tumor Formation

This review demonstrates a higher incidence of arrhythmias with intramyocardial delivery than epicardial delivery. Although safety outcomes were hardly reported, this does not preclude the possibility of arrhythmias with longer follow-up or a bigger sample size. Transplantation of hiPSC-CMs irrespective of the route of delivery or nature of construct did not cause immune rejection with single or triple immunosuppressive regimens. In cases where there was an immune response, it was mild and attenuated with time. This is relevant to the progression to clinical trials as these drugs are already used in clinical practice.

### 4.8. Preliminary Findings from Clinical Trials Show Similar Outcomes to Animal Studies, Irrespective of Construct

Four Phase I/II, open-label, clinical trials (NCT04396899, NCT04945018, NCT03763136, and NCT04696328) have investigated the therapeutic effect of hiPSC-CMs in chronic ischemic HF [66,67,68,69]. Preliminary results for seven cases have been published [67,68,69] and are represented in Table 4. Across these trials, patients had severe, symptomatic HF on maximal guideline-directed medical therapy with an NYHA Class of II/III and were eligible for CABG or heart transplant; this is an evident contrast to animal studies where populations are usually young and healthy.

Preliminary clinical trial findings concurred with preclinical results by showing improved EF, NYHA Class, evidence of reverse remodeling, and, in some cases, physical fitness. Most notably, one trial [66] showed improvements in NT-proBNP (a biomarker used to assess the degree of ventricular stretch), which is widely used in clinical practice to monitor progression and pharmacological response of HF. There were no reports of immune rejection. However, one patient (Case 5 in Table 4), found to have an increase in pre-transplantation levels of HLA-DQ, did not show improvement in all efficacy outcomes [67]. Clinical trials used the same immunosuppressive regimens as those used in animal studies. One case of atrial fibrillation and brief asymptomatic episodes of accelerated idioventricular rhythm 1–2 weeks post-transplantation was reported [66]; however, these are relatively common post-CABG arrhythmias.

## 5. Limitations of the Current Published Studies

Xenotransplantation remains a challenge in preclinical studies due to the variation in physiological and structural CM properties between humans and animals. For instance, inherent heart rates in rodent models prevent proper electromechanical coupling of human CMs and can mask arrhythmia [70]. Furthermore, animal models do not accurately reflect clinical practice where patients typically have a longstanding history of CAD.

There are distinct structural and functional differences in the coronary system between animals and humans that likely influenced this heterogeneity in outcomes [71]. The use of varied MI induction models causes heterogeneity in infarct size and depth. Cryoinjury, which involves freezing of the anterior LV wall, produces a localized infarct but does not reflect true ischemic damage and can lead to unpredictable infarct sizes and shallow infarcts (non-transmural) [71]. Moreover, collateral coronary circulation can greatly influence infarct size and myocardial recovery, which could explain the higher engraftment rates in the guinea pig models. Pigs share a similar resting heart rate to humans (50–100 bpm) and have minimal collaterals, which may augment in a chronic compensatory mechanism to ischemia [71]. In contrast, rodents have smaller arteries and a distinct septal coronary artery that courses along the right interventricular septum causing different regionality of infarction compared to large animals and humans [71]. This was seen in a guinea pig model where quantification of vascular density was unreported due to small vessel sizes [52].

Ligation of the LAD does not reflect the natural progression of coronary artery disease that occurs in humans. A drawback of this method is the inability to limit infarct expansion after the ischemic insult. An alternative to this, as seen in some studies, is the use of the ameroid constrictor and the novel intracoronary sequential sponge embolization [54], which mimics the gradual occlusion and reperfusion of the artery that is seen in humans. However, the latter can result in small infarct sizes [54]. The I/R model is clinically analogous to patients who receive timely reperfusion interventions, such as P.C.I, in clinical practice, but can cause reperfusion injury and may reduce post-MI remodeling.

There was also marked heterogeneity regarding measurement modalities, dose, and differentiation protocols. No single study explored or reported all outcomes. This lack of standardization makes it difficult to draw valid comparisons and hinders reproducibility and translation to clinical trials.

## 6. Future Directions

Even though hiPSC-CMs have already gained some traction in the clinical arena with positive preliminary results demonstrating safety and efficacy, a few key factors that guide the transition to clinical trials are yet to be considered: (1) demonstration of a similar disease pathogenesis in the animal model and (2) comparable histological phenotypic characteristics. To reduce heterogeneity and enhance reproducibility, standardization is needed. Researchers should focus on the use of porcine models with MI induction models that closely resemble the natural progression of CAD in clinical practice. Furthermore, longer follow-up durations should be pursued as clinical studies typically last for one year.

It was hypothesized that survival and viability of hiPSC-CMs would increase when transplanted outside of the inflammatory post-MI phase and during scar formation. However, the results of this review suggest that survival has less to do with incompatibility between the CMs and the host environment and more to do with their ability to structurally and functionally assimilate with the host myocardium. Therefore, researchers should focus on understanding the factors that influence survival. Most studies assessed the survival of hiPSC-CMs by histological analysis, which could only be performed after sacrifice; hence, the fate of the transplanted cells was unknown. One study [43], which showed the longest survival, used an in vivo CMR-phenotypic fate-tracking method with SPIO labeling. Previous work suggests that this method is unreliable as it cannot distinguish between dead macrophage-engulfed SPIO-labeled cells and viable ones [72,73,74]; hence, it potentially overestimates the number of viable cells at the endpoint. Research should aim to optimize modalities that track the fate of transplanted hiPSC-CMs.

Knowledge of the fetal-to-adult transition [75] of cardiomyocytes after birth could be used to develop strategies to improve post-transplantation survival of hiPSC-CMs. For instance, transcription factor GATA4 is highly expressed by cardiomyocytes in the fetal and early neonatal stages of development but is downregulated in the post-natal phase, following arrest of cell division [75]. Adult mouse cardiomyocytes overexpressing GATA4 exhibited enhanced proliferative potential and increased perfusion-dependent cardiac contractility [76]. In the post-natal phase, cardiomyocytes switch from glycolysis to oxidative phosphorylation and another transcription factor TBX20 has been shown to promote the metabolic switch to mitochondrial respiration and reduce glycolytic reserve [77]. The Hippo pathway is activated with fetal-to-post-natal cardiomyocyte transition. This inactivates its downstream effectors, YAP and TAZ, which inhibit cell division [75]. Studies show that YAP/TAZ stimulate angiogenesis via the VEGF signaling pathway [78]. Careful modulation of these transcription factors and their respective pathways can potentially advance in vivo survival of transplanted hiPSC-CMs.

Spheroids are ideal constructs due to their ischemic resistance, high purity, and ability to carry larger numbers of cardiomyocytes. However, studies used intramyocardial injections of spheroids, which increased the risk of arrhythmogenicity. This risk was not observed with epicardial constructs. Incorporation of spheroids into a patch/sheet for epicardial delivery may potentially mitigate this risk.

Pre-transplantation strategies that combine vascular-derived and adipose-derived stem cells should be implemented. More research is needed to understand the mechanisms that facilitate the potential paracrine effect of hiPSC-CMs on myocardial contractility, particularly VEGF.

## Figures and Tables

**Table 1:** General characteristics of 23 selected studies.

Author	Animal Model	Cell Type	Construct	Delivery	Days after MI	Follow-Up
Cheng et al. [32]	NHP	CMs and ECs	Cells	Intramyocardial	8 days	28 days
Kobayashi et al. [33]	NHP	CMs	Spheroids	Intramyocardial	14 days	~84 days
Lancaster J et al. [34]	Rodent	CMs and hNDFs	Patch	Epicardial	21 days	21 days
Yokoyama et al. [35]	Rodent	CMs	Patch	Epicardial	14 days	84 days
Guan X et al. [36]	Rodent	CMs	Cells	Intramyocardial	10 days	28 days
Ishida et al. [37]	Porcine	CMs	Sheets	Epicardial	30 days	60 days
Samura T et al. [38]	Rodent	CMs	Sheets	Epicardial	28 days	28 days
Zhang et al. [39]	Rodent	CMs and hADSCs	Sheets	Epicardial	14 days	28 days
Kawamura et al. [40]	Porcine	CMs	Sheets	Epicardial	28 days	56 days
Ishigami et al. [41]	Porcine	CMs/ECs/VMCs	Sheets	Epicardial	14 days	28 days
Lida J et al. [42]	Rodent	CMS	Hydrogel	Intramyocardial	7 days	7 days
Kawamura et al. [43]	Porcine	CMs	Sheets	Epicardial	28 days	~91 days
Nakazato et al. [44]	Rodent	CMs	Sheets	Epicardial	14 days	28 days
Sun X et al. [45]	Rodent	CMs	Cells	Intramyocardial	14 days	28 days
Miyagawa et al. [46]	Porcine	CMs	Patch	Epicardial	28 days	84 days
Chinyere et al. [47]	Rodents	CMs and hNDFs	Patch	Epicardial	21 days	49 days
Biagi et al. [48]	Rodent	CMs	Cells	Intramyocardial	7 days	37 days
Kawaguchi et al. [49]	Rodent/porcine	CMs	Spheroids	Intramyocardial	7/28 days	56 days
Weinberger et al. [50]	Rodent	CMs/ECs	Patch	Epicardial	7 days	28 days
Lancaster J et al. [51]	Porcine	CMs and hNDFs	Patch	Epicardial	28 days	~182 days
Querdel et al. [52]	Rodent	CMs	Patch	Epicardial	7 days	28 days
Masumoto et al. [53]	Rodent	CMs/MCs/ECs	Sheets	Epicardial	7 days	28 days
Li et al. [54]	Porcine	CM^TK+^ and CMs	Cells	Intramyocardial	84 days	84 days

NHP—nonhuman primate, EC—endothelial cell, hADSCs—human adipose derived stem cells, hNDFs—human neonatal dermal fibroblasts.

**Table 2:** Key efficacy and safety outcomes for hiPSC-CMs delivered via the intramyocardial route (spheroids, cells, and hydrogel). NM—not measured; NR—not reported. Symbol ↑ represents increase).

		Efficacy				Safety		
	Study	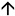 Systolic function	Reduced infarct/scar size	Survival	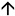 Vascular density	Arrhythmias	Immune rejection	Tumor formation
Spheroids	[33]	✓	NM	NR	NM	✓	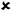	NR
	[49]	✓	✓	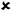	✓	✓	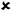	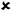
Cells	[54]	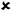	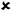	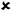	NM	✓	NR	NR
	[48]	✓	NM	✓	NM	NR	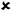	NR
	[32]	✓	✓	NR	✓	✓	NR	NR
	[36]	✓	NM	✓	NM	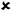	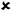	NR
	[45]	✓	✓	✓	✓	NR	NR	NR
Hydrogel	[42]	✓	NM	✓	NM	NR	NR	NR

**Table 3:** Key efficacy and safety outcomes for hiPSC-CMs delivered via the epicardial route. NM—not measured; NR—not reported; UD—undetermined (difficulty distinguishing infarct border from patch border). Symbols ↑ represents increase).

		Efficacy				Safety		
	Study	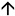 Systolic function	Reduced infarct/scar size	Survival	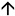 Vascular density	Arrhythmias	Immune rejection	Tumor formation
**Patches**	[34]	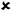	NR	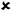	NR	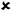	NR	NR
	[35]	✓	NM	NR	✓	NR	NR	NR
	[46]	✓	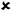	NR	✓	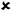	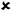	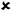
	[47]	✓	NM	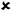	NM	NR	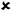	NR
	[50]	✓	UD	✓	✓	NR	NR	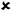
	[51]	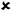	NR	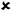	✓	NR	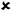	NR
	[52]	✓	NM	✓	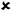	NR	NR	NR
**Sheets**	[38]	✓	NM	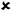	✓	NR	NR	NR
	[37]	✓	NM	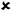	✓	NR	NR	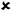
	[41]	✓	✓	✓	✓	NR	NR	NR
	[40]	✓	NM	✓	✓	NR	NR	NR
	[43]	✓	NM	✓	✓	NR	NR	NR
	[44]	✓	NM	✓	✓	NR	NR	NR
	[53]	✓	NR	✓	✓	NR	NR	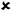

**Table 4:** Preliminary clinical outcomes and baseline characteristics. (Arrows are used for the trials that did not provide specific quantities, symbols ↑ and ↓ represent increase and decrease).

Case Number	Efficacy Outcomes	Baseline	Months Post-Transplantation			
			3	4	6	12
Case 1 (LAPiS)	LVEF (%)	26 (Echo) 15 (CMR)			28 19	
	LVEDV (mL) Echo MRI	345 431			252 389	
	NYHA Class	III			II	
	NT-proBNP (pg/mL)	11,471			5733	
Case 2 (LAPiS)	LVEF (%)	26		26		
	LVEDV (mL) Echo	324		362		
	NYHA Class	III	III			
Case 3 (LAPiS)	LVEF (%)	17	36			
	LVEDV (mL)	196	172			
	NYHA Class	III	II			
Case 4 (NCT04696328)	LVEF (%)	<30%			↑	↓
	NYHA Class	III			I	I
	LVIDs (mm)	n.a			↓	↓
	LVIDd (mm)	n.a			↓	↓
	NT-proBNP (pg/dL)	n.a			↓	↑
	Peak VO_2_ (mL/min/kg)	n.a			↑	↑
	Myocardial blood flow (mL/min/g)	n.m			1.67	3.12
Case 5 (NCT04696328)	LVEF (%)	21%			-	-
	NYHA Class	III			II	II
	LVIDs (mm)	n.a			-	-
	LVIDd (mm)	n.a			-	-
	NT-proBNP (pg/dL)	n.a			↑	↑
	Peak VO_2_ (mL/min/kg)	n.a			-	n.a
	Myocardial blood flow (mL/min/g)	0.51			0.57	0.47
Case 6 (NCT04696328)	LVEF (%)	35%			↑	↑
	NYHA Class	III			I	I
	LVIDs (mm)	n.a			↓	-
	LVIDd (mm)	n.a			↓	-
	NT-proBNP (pg/dL)	n.a			↓	↑
	Peak VO_2_ (mL/min/kg)	n.a			↓	↑
	Myocardial blood flow (mL/min/g)	1.01			0.69	0.67
Case 7 (BioVAT-HF) 46 y/o F	LVEF (%)	35%	39%			
	LVEDV (mL)	104	78			
	LVESV (mL)	68	48			

n.m—not measured; n.a—not available.

## Data Availability

No new data were created or analyzed in this study.
